# Kinetic isotope effects and how to describe them

**DOI:** 10.1063/1.4996339

**Published:** 2017-12-13

**Authors:** Konstantin Karandashev, Zhen-Hao Xu, Markus Meuwly, Jiří Vaníček, Jeremy O. Richardson

**Affiliations:** 1 Laboratory of Theoretical Physical Chemistry, Institut des Sciences et Ingénierie Chimiques, Ecole Polytechnique Fédérale de Lausanne (EPFL), CH-1015 Lausanne, Switzerland; 2 Department of Chemistry, University of Basel, Klingelbergstrasse 80, CH-4056 Basel, Switzerland; 3 Laboratory of Physical Chemistry, Department of Chemistry and Applied Biosciences, Eidgenössische Technische Hochschule Zürich (ETHZ), CH-8093 Zürich, Switzerland

## Abstract

We review several methods for computing kinetic isotope effects in chemical reactions including semiclassical and quantum instanton theory. These methods describe both the quantization of vibrational modes as well as tunneling and are applied to the ⋅H + H_2_ and ⋅H + CH_4_ reactions. The absolute rate constants computed with the semiclassical instanton method both using on-the-fly electronic structure calculations and fitted potential-energy surfaces are also compared directly with exact quantum dynamics results. The error inherent in the instanton approximation is found to be relatively small and similar in magnitude to that introduced by using fitted surfaces. The kinetic isotope effect computed by the quantum instanton is even more accurate, and although it is computationally more expensive, the efficiency can be improved by path-integral acceleration techniques. We also test a simple approach for designing potential-energy surfaces for the example of proton transfer in malonaldehyde. The tunneling splittings are computed, and although they are found to deviate from experimental results, the ratio of the splitting to that of an isotopically substituted form is in much better agreement. We discuss the strengths and limitations of the potential-energy surface and based on our findings suggest ways in which it can be improved.

## I. INTRODUCTION

Kinetic isotope effects (KIEs) are for many chemical processes a useful experimental observable which can be used alongside theoretical analysis to provide information on the mechanisms involved. The most common example is that the rate of a process decreases on replacing a hydrogen atom with a deuterium. If molecules moved purely with classical mechanics, only very small KIEs would be seen, due to a frequency shift, but these are much enhanced by the zero-point energy (ZPE) effects of quantum mechanics. If there is a potential barrier to the transfer of a hydrogen atom, then tunneling can also play a large role at low temperatures leading to the largest KIEs. Large kinetic isotope effects relating to hydrogen transfer of up to 700 have been observed in enzyme catalysis,^1^ although the mechanism for the process is still debated.^2^

In order to describe these effects within an atomistic simulation, it is necessary to go beyond classical molecular dynamics (MD) and instead use approaches based on imaginary-time path integrals.^3–5^ In this short review, we therefore first compare semiclassical (SCI)^6,7^ and quantum instanton (QI) methods^8,9^ and also the related ring-polymer molecular dynamics (RPMD) approach.^10,11^ They are tested on hydrogen transfers in a selection of small systems for which exact results are available for comparison so that the relative errors of each approach can be compared more closely.

While the examples of H + H_2_ and H + CH_4_ allow the treatment of the nuclear dynamics using explicit, high-level [MP2 or CCSD(T)] on-the-fly quantum methods for the electronic structure to investigate the KIE, this is usually not possible for larger, biologically relevant systems. For such applications, it is important to have meaningful representations of the intermolecular interactions that allow simulations of large systems and for extended time scales. As a step in this direction, we use malonaldehyde (MA) as an example because for this system, a high-accuracy CCSD(T) PES exists and various relevant observables have been determined that can serve as benchmarks.^12^ Such data can be compared with a more approximate molecular mechanics with proton transfer (MMPT) treatment using an empirical force field that allows bond-breaking and bond-formation and long-time simulations. A representation inspired by and based on the principles underlying empirical force fields can also be employed for simulations in biological systems, and comparison with more accurate treatments allows one to delineate the range of validity and possible future improvements of such more approximate methods. This information will be of great value in biomolecular simulations of KIEs, which serve as an important observable to understand the contribution (or absence thereof) of zero-point and/or tunneling effects to protein catalysis.^1,13^

Another well-studied system exhibiting a KIE is that of the Azzouz-Borgis model of proton transfer in solution.^14–16^ We briefly review some of the approaches which have been applied to this system, which remains a serious challenge to quantum dynamics methods.

In each example, we highlight the cause of the kinetic isotope effect, be it due to tunneling or zero-point energy effects, and compare the accuracy of the different methods in the high and low temperature limits. We discuss which factors must be well described in order to obtain accurate predictions for the KIE, and also for the absolute values of the rate or tunneling splitting.

## II. THEORY

Several approaches are currently used for calculating rate constants in situations where quantum effects are not negligible. One approach consists of adding a tunneling correction to transition-state theory,^17,18^ others approximate the quantum propagator either by treating it semiclassically^13,19^ or by treating only one or two degrees of freedom quantum mechanically.^20^ Another promising method is ring-polymer molecular dynamics,^11,21,22^ which can capture both classical recrossing and many quantum effects. In this review, we concentrate on two related methods also based on imaginary-time path integrals, namely, the semiclassical and quantum instanton theories.

### A. Semiclassical instanton theory

One of the most efficient methods for simulating quantum nuclear effects in molecular systems is semiclassical instanton (SCI) theory.^6,23^ The approach can be used either to compute tunneling splittings or the rate constant for a reaction through an energy barrier.^24,25^ These methods can be rigorously derived from steepest-descent approximations to a path-integral description of the tunneling process.^7^ Instanton rate theory is a generalization of transition-state theory, which includes delocalization, tunneling, and zero-point energy effects.

There are a few versions of semiclassical instanton theory discussed in the literature.^26^ Most of them are in principle exactly equivalent, as shown in Refs. 7 and 27, although differences exist in the practical implementation. The instanton trajectory is defined as a classical periodic orbit in the upside-down potential.^6^ In previous work, the instanton pathway has been obtained by “shooting”^23,28^ or by optimizing the trajectory in a normal-mode expansion.^26^ However, finding such orbits using classical dynamics can be numerically extremely difficult, as the trajectories are unstable.^28^ An alternative approach is much more efficient and searches for a stationary point in the extended ring-polymer space.^25,29^ The optimized ring-polymer configuration is equivalent to a discretization of the instanton trajectory with equal imaginary-time-steps. In this work, we employ this approach, known as the ring-polymer instanton method.^25,29^ The algorithms employed are given in detail in Ref. 30.

The procedure for obtaining the ring-polymer instanton rate is closely related to the commonly used Eyring approach for classical transition-state theory. In both cases, the translational, rotational, and vibrational partition functions for the reactants are compared with those at the transition state. The major difference is that at low temperatures, the saddle point is no longer identified as the transition state for the reaction, but instead a stretched ring-polymer configuration, known as an instanton, is used. This leads to an effective activation energy, which is lower than the barrier top, thus accounting for the speed-up of the reaction rate due to tunneling.^31^

The rigid-rotor and harmonic approximations are made to describe the reactant partition function and the equivalent approximations are made for the modes perpendicular to the instanton trajectory. However, along the instanton trajectory, the anharmonicity and curvature of the potential-energy surface are described in full. For this reason, the semiclassical instanton approximation captures the most important quantum dynamics effects on the rate. Although the harmonic approximation introduces errors into the reactant partition function,^32^ some error cancellation will occur when it is taken in a ratio with the instanton partition function computed with the same approximation.

Typically, the total computational cost to locate the instanton and compute the rate at a given temperature is about 10–100 times greater than that of a standard transition-state search and frequency calculation. As the ring-polymer instanton method requires calculations only of a small number of configurations in the barrier region, it is feasible to utilize expensive *ab initio* computations of the potential-energy surface on the fly. This makes good practical sense, since often the errors in the PES dominate a rate calculation,^33^ making it more important to ensure a high level of accuracy in the electronic-structure calculations rather than the nuclear dynamics.

The ring-polymer instanton method (also referred to as harmonic quantum transition-state theory^29^) has been used to compute tunneling rates for a wide range of reactions using either fitted potential-energy surfaces or density functional theory.^29,34–42^ Kinetic isotope effects have also been previously studied with this approach^40,43,44^ with some interesting implications for astrochemistry.^45^ Tunneling splittings have been computed for various molecules and molecular clusters, usually describing a proton transfer or hydrogen-bond rearrangement.^46–50^ The isotope effect on the tunneling splittings due to deuteration of water clusters can be more than 100,^47,51^ whereas substituting ^16^O for ^18^O causes only a 15% reduction, which nonetheless can be measured and is in excellent agreement with the theoretical prediction.^49^

Instanton calculations of ground-state tunneling splittings^24^ are performed in the zero-temperature limit and thus require more ring-polymer beads to achieve convergence than a rate calculation. For these reasons, most applications have used potential-energy surfaces fitted to high-level *ab initio* calculations rather than obtaining the potential on the fly. The SCI method provides an excellent test of the accuracy of the surface when the computed tunneling splitting is compared with the experimental value.^52^ Instanton theory is valid for computing small tunneling splittings but shows significant errors when the splitting becomes large relative to the vibrational-state spacing,^24^ and in this way complements the diffusion Monte Carlo approach^53,54^ for which statistical errors can make estimating small splittings difficult.^52^ For instance, on the PES of Wang *et al.*,^12^ which is a fully dimensional PES fitted to CCSD(T) calculations, the instanton approach predicts the splitting of malonaldehyde to be 25 cm^–1^.^50^ For comparison, an exact diffusion Monte Carlo calculation on the same PES gives a tunneling splitting of 21–22 cm^–1^ depending on the coordinate system used.^12^

Unlike exact quantum dynamics, the instanton approach^24^ scales well with system size and is able to simulate molecules and clusters in full dimensionality. A recent application to the double proton transfer in the formic acid dimer gave good agreement with experiment again showing only about a 20% error.^52^ A systematic study of the dependence of the tunneling splitting on the number of degrees of freedom included in the calculation shows that the results are very sensitive and require almost all modes to be included before converging.

The SCI approach is only appropriate below a certain cross-over temperature, given approximately by

$$T_{c}=\frac{\hbar \bar{\omega }}{2\pi k_{B}},$$ (1)

where $\bar{\omega }$ is the absolute value of the imaginary frequency at the saddle point. Above this temperature, tunneling is no longer dominant and the reaction rate can be computed using classical rate theory, possibly including shallow tunneling corrections such as Wigner's second order expression^17^

$$\kappa =1+{\left(\hbar \beta \bar{\omega }\right)}^{2}/24,$$ (2)

which multiplies the classical Eyring formula *k*_TST_ to give an approximation to the quantum rate $k\approx \kappa k_{\text{TST}}$.

The SCI method is related to approaches such as the small (SCT) and large (LCT) curvature tunneling approximations.^55^ These are, however, limiting cases of the instanton for which the tunneling pathway is determined *a priori* and not optimized as in the instanton approach. Likewise, the so-called optimized multidimensional tunneling approximation (OMT) simply chooses between the SCT and the LCT and is not optimized in the sense of the instanton trajectory. This provides a simple scheme which has worked well in practice in many situations. However, in general, the instanton approach will always give a better description of the tunneling process, especially at low temperatures as has been seen in previous studies.^29^ There are important examples of tunneling pathways, such as those in water clusters,^47,49^ where it was necessary to employ the instanton approach to understand the mechanism rather than relying on the simpler approximations.

### B. Quantum instanton approximation

The instanton approach described in Sec. II A is a quantum generalization of transition-state theory. Among other such quantum transition-state theories^2,56^ which take nuclear quantum effects into account is the quantum instanton (QI) approximation.^8^ This is motivated by the SCI theory^6,28,57^ and, as the name suggests, takes into account only the zero-time properties of the reactive flux–flux correlation function.^58^ However, in contrast to the semiclassical instanton, the quantum instanton approximation does not employ a steepest-descent approximation in the coordinate space and treats the Boltzmann operator exactly quantum mechanically. This improvement makes quantum instanton quite accurate as verified in many previous applications of the method.^59–64^

In the QI approximation, the thermal rate constant *k* at temperature *T* is expressed as

$$k_{\text{QI}}(T)=\frac{\sqrt{\pi }}{2}\frac{\hbar C_{\text{ff}}\left(0\right)}{Q_{r}\Delta H},$$ (3)

where *Q_r_* is the reactant partition function, $C_{\text{ff}}\left(t\right)$ is the flux–flux correlation function at time *t*, which measures the correlation between a flux at time 0 through a dividing surface *a* and flux at time *t* through a dividing surface *b*. Finally, ΔH is a certain type of energy variance that is obtained from the delta–delta correlation function $C_{\text{dd}}(t)$ and the dividing surfaces are determined in such a way that $C_{\text{dd}}(0)$ is a saddle point with respect to the variation of their positions.^8,65^ Like the SCI approximation, the QI approximation also takes into account zero-point energy and quantum-mechanical tunneling, but it does not assume separability of rotations and vibrations, harmonic approximation for vibrations, or the rigid-rotor approximation for rotation. When implemented using Feynman path-integral formulation of quantum mechanics, the QI takes into account all tunneling paths and not only the most important “instanton path” used in the SCI approximation.

The QI approximation can be used to compute absolute rates, but this is fairly expensive due to the necessity of sampling many path-integral configurations to converge the ratio of $C_{\text{dd}}(0)/Q_{r}$, and also due to the search of the optimal dividing surfaces. Because of this, at the moment, on-the-fly *ab initio* evaluation of *k*_QI_ appears out of reach, or at least not very practical. On the other hand, it is generally much easier to compute the KIEs, which are ratios of rate constants, and therefore can be written as a product of several ratios

$$\frac{k_{\text{QI}}^{\left(A\right)}}{k_{\text{QI}}^{\left(B\right)}}=\frac{Q_{r}^{\left(B\right)}}{Q_{r}^{\left(A\right)}}\frac{\Delta H^{\left(B\right)}}{\Delta H^{\left(A\right)}}\frac{C_{\text{dd}}^{\left(A\right)}\left(0\right)}{C_{\text{dd}}^{\left(B\right)}\left(0\right)}\frac{C_{\text{ff}}^{\left(A\right)}\left(0\right)/C_{\text{dd}}^{\left(A\right)}\left(0\right)}{C_{\text{ff}}^{\left(A\right)}\left(0\right)/C_{\text{dd}}^{\left(B\right)}\left(0\right)},$$ (4)

where the quantities ΔH and $C_{\text{ff}}\left(0\right)/C_{\text{dd}}\left(0\right)$ can be evaluated directly by path-integral sampling, while the ratios $Q_{r}^{\left(B\right)}/Q_{r}^{\left(A\right)}$ and $C_{\text{dd}}^{\left(B\right)}(0)/C_{\text{dd}}^{\left(A\right)}(0)$ can be evaluated easily via the thermodynamic integration with respect to mass.^9,66,67^ Although the QI is very accurate at low temperatures, this approximation, as any other quantum transition-state theory, cannot take into account classical recrossing, which is typically more important at high temperatures. Moreover, the QI absolute rate expression (3) overestimates the rate constant by a factor $\sqrt{\pi /2}\approx 1.25$ at high temperatures, which can, however, be easily fixed with an *ad hoc* correction.^8^ Moreover, even without the *ad hoc* correction, this factor does not play a role in evaluating KIEs because it approximately cancels between the two isotopologues *A* and *B*, and, therefore, the high temperature limit of the QI approximation, Eq. (4), agrees with the high temperature limit of classical transition-state theory for the KIEs.

### C. Evaluation of kinetic isotope effects with an accelerated version of the quantum instanton approximation

Quantum instanton theory expresses the reaction rate in terms of imaginary-time correlation functions, which in turn can be evaluated by path integral Monte Carlo (PIMC) methods.^65^ A drawback common to all path-integral methods is that they operate in an effective configuration space of greatly increased dimensionality, leading to high computational cost. Indeed, the quantum limit is approached only as the number of dimensions goes to infinity. Several strategies have been proposed to bypass the problem; here, we demonstrate how two such approaches allow accelerating quantum instanton calculations of the kinetic isotope effects.

The first approach employs Boltzmann operator factorizations of higher order of accuracy. The resulting path-integral representations of relevant quantities exhibit faster convergence to the quantum limit, allowing a reduction of the effective dimensionality of the calculation.^68–73^ The second approach uses improved estimators with lower statistical errors, which permit shortening the Monte Carlo simulation.^66,74^ In Ref. 67, these two strategies were combined; the resulting method was tested on the model $\cdot H_{\alpha }{+H}_{\beta }H_{\gamma }\to H_{\alpha }H_{\beta }+\cdot H_{\gamma }$ rearrangement and on the reaction ${\text{CH}}_{4}+\cdot H\to \cdot {\text{CH}}_{3}{+H}_{2}$, a process whose kinetic isotope effects have been studied in detail both experimentally and theoretically, with classical transition-state theory, several of its corrected versions,^75,76^ reduced dimensionality quantum dynamics,^77^ and RPMD.^78^

To quantify the gain due to the accelerated quantum instanton methodology, in Table I, we compare the speedups achieved by different combinations of path-integral factorizations and estimators. The speedups were estimated as the relative computational times needed to converge the kinetic isotope effect within an overall 2% discretization and statistical error. (More precisely, the speedup values were estimated as the relative computational times needed to obtain the kinetic isotope effect with a 1% discretization and 1% statistical errors; these were estimated by running several test calculations with different numbers of imaginary-time slices, *P*, and by assuming that the cost of a calculation aiming to reproduce the target statistical and discretization errors is approximately proportional to the required value of *P* and to the square of the statistical error observed in the test calculation for this *P*. (For details, see Ref. 67.) The table shows that the accelerated methodology can obtain the results with the same accuracy as much as 100 times faster than the original path-integral implementation from Ref. 9.

**TABLE I.** Estimated speedups of the quantum instanton calculations of the kinetic isotope effect ⋅H + H_2_/⋅D + D_2_ at 200 K achieved by the use of various combinations of path-integral factorizations and estimators (th = thermodynamic, v = virial). Speedup “1” (i.e., no speedup) corresponds to the standard method employing a combination of the Lie-Trotter factorization and thermodynamic estimators. The Trotter numbers *P* required for each factorization are shown as well.

	Lie-Trotter (*P* = 128)		Suzuki-Chin (*P* = 40)	
Factorization	th	v	th	v
Speedup	1	34	12	97

## III. BIMOLECULAR H TRANSFER

### A. Eckart barrier

The Eckart barrier,^79^ $V(x)=V^{‡}{\text{sech}}^{2}(x/a)$, is a one-dimensional system which provides the simplest model of a reaction with a barrier; it has been used extensively as a test system for reactive dynamics methods, as its transition probability is analytically available, making it trivial to recover the exact reaction rate. Moreover, the lack of classical recrossing in this system allows to focus on errors due to quantum-mechanical effects. Instead of the absolute rate of the reaction across the Eckart barrier, one therefore often considers only the tunneling correction, i.e., the ratio of the exact quantum and classical rates, because the ratio depends on only two dimensionless parameters. The first is the “degree of quantumness” $\alpha =\pi \sqrt{2ma^{2}V^{‡}}/\hbar$, while the second one is the ratio $\beta /\beta_{c}$, where $\beta_{c}=\alpha /V^{‡}$ is the inverse crossover temperature. Our calculations used $\alpha^{\left(H\right)}=12$, as in Ref. 80, which models an ⋅H + H_2_ reaction. The rates were compared for a range of values of the reduced temperature $\beta /\beta_{c}^{\left(H\right)}$. Analytic results are available for the exact microcanonical reaction probability^79^ and the semiclassical instanton rate constant,^31^ but the QI results are computed numerically.

Several tunneling corrections and kinetic isotope effects, corresponding to the doubling of the particle mass, *m*, without changing *V*(*x*) were evaluated with both the semiclassical and quantum instanton formalisms and are presented in Table II along with the exact quantum values. Note that $\beta_{c}^{\left(H\right)}$ refers to the crossover temperature of the system with the lighter mass and that although it is different for the system with the heavier mass, the same value of *β* was used for both when computing the KIE. For the tunneling corrections at higher temperatures, one can see that the quantum instanton value displays its characteristic ∼25% error (since the *ad hoc* correction was not employed and since there is no recrossing), while the standard semiclassical instanton is not defined at temperatures above the crossover temperature.^31^ At lower temperatures, however, both semiclassical and quantum instanton formalisms reproduce the tunneling correction quite accurately, with quantum instanton performing slightly better in the considered temperature range except for the large statistical fluctuations at the lowest temperature. As for the kinetic isotope effects, a large cancellation of errors is observed. For the quantum instanton approximation, the cancellation of systematic errors helps at high temperatures, whereas at low temperatures, the direct calculation of the KIE (without computing the absolute rates) reduces statistical errors. Both semiclassical and quantum instanton methods reproduce the exact quantum values of the KIEs very accurately.

**TABLE II.** Semiclassical instanton (SCI), quantum instanton (QI), and exact quantum mechanical (QM) values of the tunneling correction, *k*/*k*_TST_, and the kinetic isotope effect corresponding to a double increase of the mass for an Eckart barrier. Numbers in parentheses denote powers of ten.

$\beta /\beta_{c}^{\left(H\right)}$	Tunneling correction			Kinetic isotope effects		
	SCI	QI^81^	QM	SCI	QI^81^	QM
0.5	⋯	1.91	1.58	⋯	1.74	1.77
1	⋯	7.49	6.16	⋯	3.42	3.48
2	3.50(3)	4.35(3)	4.29(3)	5.15(1)	5.37(1)	5.70(1)
4	3.27(12)	(3.89 ± 0.03) (12)	3.96(12)	1.03(3)	1.03(3)	1.08(3)
8	3.62(32)	(5.0± 0.5) (32)	4.08(32)	4.63(3)	4.44(3)	4.58(3)

### B. ⋅H + H_2_

The simplest thermally activated reaction, $\cdot H_{\alpha }+H_{\beta }H_{\gamma }\to H_{\alpha }H_{\beta }+\cdot H_{\gamma },$ exhibits a large deviation from the Arrhenius law and large kinetic isotope effects due to both tunneling and zero-point energy effects, and hence provides an excellent test case for approximate methods,^42,82,83^ in particular since the exact results are again available.^84^

In Table III, we compare rates from the SCI approximation with the exact results for two isotopic variants of this reaction. The BKMP2 potential-energy surface^85^ was used by all three methods. The instanton rates presented are converged with respect to the number of beads to two significant figures. It was necessary to use 512 beads at the lowest temperature studied.

**TABLE III.** Semiclassical instanton (SCI) and exact quantum-mechanical (QM) values of $\cdot {H+H}_{2}$ and $\cdot {D+D}_{2}$ rates in cm^3^/s. The crossover temperatures are 345 and 244 K for the reactions.

T/K	⋅H + H_2_		⋅D + D_2_	
	SCI	QM	SCI	QM
300	5.2(−16)	3.24(−16)	⋯	4.65(−17)
250	4.3(−17)	3.67(−17)	⋯	3.53(−18)
200	2.2(−18)	2.01(−18)	1.2(−19)	8.92(−20)

The instanton approach is seen to agree within about 20% except for the highest temperature studied in each case when the rate is overestimated by a larger factor. It is a well-known effect which causes the SCI to overestimate the rate near to the crossover temperature by about a factor of two. This could be corrected by a number of proposed extensions to the approach,^26^ including an explicit integration over microcanonical results.^31^ The related approaches of RPMD and QI also avoid this problem.

Table IV compares the values of the kinetic isotope effect ⋅H + H_2_/⋅D + D_2_ obtained with the Wigner-Eyring TST, SCI, QI, and exact quantum-mechanical calculation from Ref. 9. At low temperatures, where the quantum effects are largest, the error of the quantum instanton is, remarkably, below 5%. At high temperatures, the error is somewhat larger, but this is expected since the quantum instanton approximation, as any other classical or quantum transition-state theory, neglects recrossing effects. These become more pronounced at high temperatures, and are therefore more important for D + D_2_, which reaches this regime before H + H_2_. The recrossing, which is a purely classical effect, can be captured easily by classical or ring-polymer molecular dynamics simulations.

**TABLE IV.** Kinetic isotope effect $\cdot {H+H}_{2}/\cdot {D+D}_{2}$ at different temperatures obtained with the semiclassical instanton (SCI), quantum instanton (QI) approximation, and with an exact quantum-mechanical (QM) method.

*T* (K)	Wigner-Eyring	SCI	QI	QM^a^	% error (QI)^b^
2400	1.57	⋯	1.55 ± 0.01	⋯	⋯
1500	1.78	⋯	1.81 ± 0.01	2.27	–20
1000	2.17	⋯	2.23 ± 0.01	2.61	–15
600	3.11	⋯	3.29 ± 0.02	3.42	–4
400	4.37	⋯	4.87 ± 0.03	4.74	3
300	5.60	⋯	7.35 ± 0.05	6.97	5
250	6.52	⋯	9.92 ± 0.09	10.40	–5
200	7.82	19.4	22.6 ± 0.3	22.53	<1

^a^ From Ref. 9.

^b^ The error is defined as $({\text{KIE}}_{\text{QI}}-{\text{KIE}}_{\text{QM}})/{\text{KIE}}_{\text{QM}}\times 100\%$.

At high temperatures, besides differences in recrossing for H and D, this kinetic isotope effect is mainly due to zero-point energy effects, which are well-described by Wigner-Eyring TST, which therefore gives results similar to those of QI. This suggests that in this case the rigid-rotor harmonic approximation is fairly good, as would be expected, as there are no low frequency floppy modes in this system. At lower temperatures, tunneling starts to become the dominant effect contributing to the KIE. This is only approximately described by Wigner-Eyring TST and the error increases as the temperature is lowered. A much better description of the tunneling for lower temperatures is given by SCI, which has a fairly good agreement with the QM results at 200 K. This approach cannot currently be used at higher temperatures, which would be above the crossover temperature for D + D_2_.

### C. ⋅H + CH_4_

**FIG. 1.**
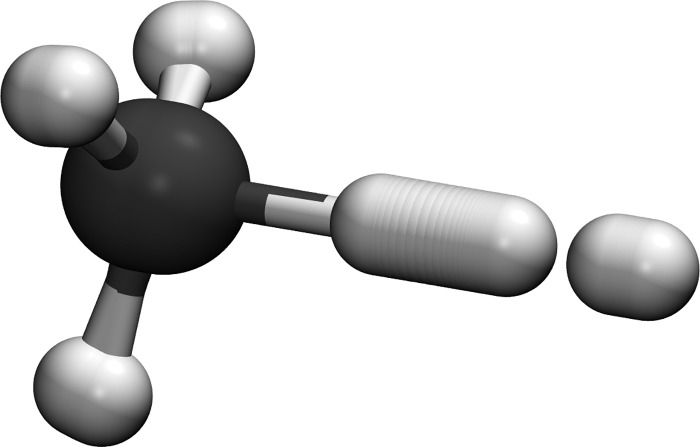
Representation of the ring-polymer instanton describing proton tunneling in the $\cdot {H+\text{CH}}_{4}$ reaction at 200 K. All atoms take part to some extent in the tunneling process and become delocalized as they pass through the potential barrier.

The ring-polymer instanton method has been implemented in the Molpro electronic-structure package,^86,87^ which allows the rates to be computed on the fly, without the need for a fitted analytic potential-energy surface. This has been applied to the ⋅H + CH_4_ reaction (see Fig. 1), using coupled-cluster methodology, showing how the efficiency of the instanton method allows the user to systematically converge the tunneling rate with respect to the level of electronic-structure theory.^30^

Testing RCCSD-F12 and RCCSD(T)-F12 with both cc-pVDZ and cc-pVTZ basis sets, barrier heights were found which varied from 63.21 kJ mol^–1^ with the most accurate combination to 67.51 kJ mol^–1^ for the least accurate. This variation in barrier height can cause more than a 10-fold difference in the predicted rate constant at 200 K, as is easily estimated from the Arrhenius equation. However, it is not just the barrier height which affects the quantum rate, but also the barrier shape. For instance, a thinner barrier is more conducive to tunneling. It was also found that the tunneling factor predicted by the lower level of theory was up to a factor of 4 larger than that predicted by the higher level. At 200 K, the best estimate of the tunneling factor is about 700, and it is thus clear that the inclusion of quantum effects in low-temperature rate calculations cannot be ignored.

In Table V, we compare the instanton rates computed on the fly with a high-quality RCCSD(T)-F12/cc-pVTZ electronic structure calculations with rates on the CBE potential surface.^90^ All instanton rates presented are converged with respect to the number of beads, for which it was necessary to use at least 256 for the lowest temperature. Also given for comparison are the rates found from exact quantum dynamics on this same surface,^88^ and from the supposedly more accurate WWM surface.^89^

**TABLE V.** Computed rates for $\cdot {H+\text{CH}}_{4}$ in cm^3^/s. The *ab initio* instanton results are from Ref. 30, the MCTDH/CBE results from Ref. 88, and MCTDH/WWM from Ref. 89.

T/K	SCI/*ab initio*	SCI/CBE	MCTDH/CBE	MCTDH/WWM
300	1.7(−19)	1.8(−19)	8.4(−20)	7.8(−20)
250	4.8(−21)	4.2(−21)	3.1(−21)	3.6(−21)
200	1.1(−22)	5.7(−23)	⋯	⋯
150	1.8(−24)	4.6(−25)	⋯	⋯

First, we compare the SCI results with the exact MCTDH approach on the CBE surface. As expected, the SCI result overestimates the rate at 300 K by about a factor of 2, as this is just below crossover, but there is fairly good agreement (∼35% error) at 250 K. This agreement would be expected to continue to lower temperatures but unfortunately, no MCTDH results are available, as the method becomes more computationally demanding in this regime.

A comparison between the *ab initio* instanton results and those on the fitted surface shows good agreement at high temperatures, but the error increases as the temperature drops. Assuming that the *ab initio* PES is correct, this discrepancy can be assigned to errors in the fit of the CBE surface. A similar effect on the rate caused by these errors is seen for MCTDH calculations on using the more accurate WWM surface instead of CBE. These errors have a larger effect on the low temperature instantons, which are more delocalized and sample a larger region around the barrier. In fact, at 250 K, the error caused by the fit is similar in magnitude to that assigned to the instanton approximation.

It is not currently possible to perform exact quantum dynamics in full dimensionality using on-the-fly *ab initio* calculations for this system, although some progress has been made toward this goal.^91^ Therefore, in order to study such chemical reactions, it is necessary to make at least one approximation. This application shows that at low temperatures, the accuracy of the *ab initio* SCI approach is similar to that of using exact quantum dynamics approaches with the CBE fitted PES. For systems for which an accurate fitted potential-energy surface is not available or for which exact quantum dynamics becomes too expensive, on-the-fly implementations of instanton theory are expected to give the most reliable estimates of the tunneling rate.

The CVT/*μ*OMT method on the CBE PES gives a rate of 1.10 × 10^–19 ^cm^3^/s at 300 K and 4.3 × 10^–21 ^cm^3^/s at 250 K.^92^ These rates are not so different from the instanton results, but we expect that larger deviations will degrade them at lower temperatures as has been shown for this system on a different PES.^29^ This is because at lower temperatures, more corner cutting takes place,^93^ which is not so well described by CVT/*μ*OMT.

In Table VI, we compare KIEs obtained using the CBE potential-energy surface^90^ and different approaches. Unfortunately, MCTDH results on the CBE surface are not available for the deuterated reaction, and so, there is no exact benchmark for this KIE. Results do exist for MCTDH^89^ on the WWM surface,^94,95^ but it was not possible to obtain SCI and QI results on this surface, as this is built using a Shepard interpolation scheme and has some bumps causing sharp variations in the first and second derivatives. This does not cause problems for the MCTDH method, but makes the steepest-descent approximation of the SCI approach invalid and forbids the use of the acceleration schemes for QI.

**TABLE VI.** Comparison of the kinetic isotope effects $\cdot {H+\text{CH}}_{4}/\cdot {D+\text{CH}}_{4}$ evaluated with semiclassical instanton (SCI), quantum instanton (QI) and ring-polymer molecular dynamics (RPMD)^78^ on the CBE potential-energy surface.^90^

T/K	SCI	QI^81^	RPMD
700	⋯	0.79 ± 0.01	0.80
500	⋯	0.64 ± 0.01	0.65
400	⋯	0.54 ± 0.01	⋯
300	0.38	0.42 ± 0.01	0.46
250	0.30	0.37 ± 0.01	⋯
200	0.24	0.35 ± 0.02	0.30
150	0.16	0.32 ± 0.07	⋯

Above the crossover temperature, ring-polymer molecular dynamics and quantum instanton approaches yield almost identical KIEs, which shows that recrossing cannot be an important factor here. Below the crossover temperature, all three approximate methods give similar results. Ring-polymer molecular dynamics and quantum instanton both account for anharmonicities of the potential in directions perpendicular to the reaction coordinate and thus one would expect them to yield more accurate results.

Benchmark results for MCTDH on CBE for the isotopically substituted reaction would be needed to resolve the issue of which of the methods is most accurate in this case. This highlights the need for benchmark MCTDH results for isotopically substituted reactions on smooth PESs, which will help the development of improved approximate quantum dynamics methods.

The SCI approach makes it possible to determine the cause of the KIE in a simple way. In Table VII, we separate the KIE into four contributions which multiply together to give the total result in the final column.^30^ The translational contribution (trans.) is independent of temperature, and the rotational contribution (rot.) only varies by a small amount. The ratio of the exponentials (exp.) of the action describes the contribution of tunneling to the KIE, which as expected, is more important at lower temperatures and decreases the rate of the ⋅D + CH_4_ reaction relative to ⋅H + CH_4_. However, this effect is outweighed by the contribution of the vibrations (vib.), which causes an inverse KIE due to the lower frequencies experienced by the D atom.

**TABLE VII.** Contributions to the SCI kinetic isotope effect for $\cdot {H+\text{CH}}_{4}/\cdot {D+\text{CH}}_{4}$ on the CBE potential-energy surface.^90^

T/K	Trans.	Rot.	Vib.	Exp.	KIE
300	2.59	0.67	0.211	1.02	0.38
250	2.59	0.68	0.152	1.12	0.30
200	2.59	0.68	0.095	1.42	0.24
150	2.59	0.68	0.042	2.20	0.16

The reason that tunneling is not the dominant contribution to the KIE is because in this case, it is the H atom leaving the methane molecule which has the largest displacement along the instanton trajectory (see Fig. 1) and this is not affected by the isotopic substitution. A very different KIE would be seen by, for example, ⋅H + CD_4_.^67^

## IV. MALONALDEHYDE

In order to characterize the properties of a shared proton between an acceptor (A) and donor (D) moiety, various experimental methods have been used in the past. One of the most successful approaches is based on optical spectroscopy,^96–100^ which provides valuable benchmarks for computational studies.

**FIG. 2.**
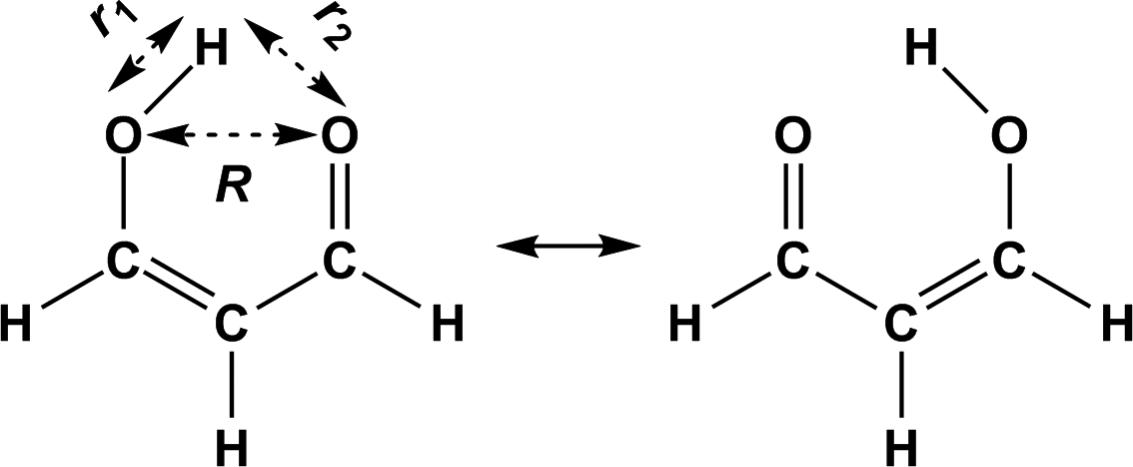
Intramolecular proton transfer of malonaldehyde in enol forms. Both the minimum and transition-state geometries are planar.

Malonaldehyde (MA) exhibits an intramolecular hydrogen bond (see Fig. 2) and has long served as a typical proton/hydrogen transfer system to test and validate various computational approaches. This makes MA an ideal molecule to validate more approximate treatments of the nuclear dynamics and intermolecular interactions, which are suitable for application to large biomolecular systems on extended time scales. This is of particular relevance if insight into the importance and nature of quantum contributions (zero-point and tunneling) to protein catalysis is sought.^101–103^ Hence, comparison of rigorous calculations with more approximate treatments of the intermolecular interactions and the nuclear dynamics appears to be a meaningful way forward to delineate the validity of such methods. Also, potential shortcomings of such simplifications and how to further improve the treatments can be studied by comparing with results based on more rigorous approaches. For MA, a high-accuracy CCSD(T) PES exists,^12^ which provides benchmarks for such validations. Experimentally, the ground state tunneling splitting was determined to be 21.58314 cm^–1^ by different experiments^104,105^ and infrared spectra are available for comparison.^106–109^

Molecular Mechanics with Proton Transfer (MMPT) is a force field-based method, which allows bond formation and bond breaking between the transferring hydrogen atom and the acceptor or donor atom, respectively.^110^ Originally, it was developed to study the nature and assignment of infrared spectroscopic features in the gas and condensed phase and for computing scalar NMR coupling constants.^111–113^ These examples highlight that different parts of the PES and different characteristics (e.g., couplings) are sensitive to particular experimental observables.^114^ In other words, when considering a range of observables each of which probes different regions and properties of a PES (such as the well depth, local curvature, or coupling between different degrees of freedom), a quantitative description of all these observables becomes very challenging already for moderately large systems.

In MMPT, a multi-dimensional PES for the proton motion is parametrized from *ab initio* calculations and fit to efficient representations based on Morse potentials. The reference data^110^ were obtained at the MP2 level of theory and the fitting error over the entire grid is well below 1 kcal/mol (350 cm^–1^), but, of course, not of spectroscopic accuracy. As mentioned above, further adaptation can be achieved through morphing.^98,100,114,115^ The additional MMPT-energy is written as

$$V_{\text{MMPT}}=\{\begin{array}{cc} V_{0}(R,\rho )+k\cdot \theta^{2},\;\text{for}\;a\;\text{linear}\;H-\text{bond} & \\ V_{0}(R,\rho )+V_{d}(R,\rho ,d),\text{for}\;a\;\text{non}-\text{linear}\;H-\text{bond}, \end{array}$$ (5)

where *R* is the distance of D and A, and *r* is the distance of D and H*. These two variables are combined in a progression coordinate *ρ* defined as $\rho =(r-r_{0})/(R-R_{0})\in [0,1]$ with *r*_0_ = 0.8 Å and *R*_0_ = 1.6 Å. In Eq. (5), the 2D potential $V_{0}(R,\rho )$ is a superposition of Morse potentials. For MA, the parametrization of the MMPT force field has been validated by comparing with the experimental infrared spectra.^116^ The minimum and transition-state geometries of the MMPT force field are planar in agreement with *ab initio* calculations.

Such a combination of an explicitly parametrized, fully anharmonic three-dimensional PES with a conventional force field is computationally efficient and adaptable, but has also potential shortcomings. For example, the coupling between the high-accuracy representation and the conventional force field is not straightforward to control. Although the electronic barrier for proton transfer may be accurately described, the zero-point-energy corrected barrier height depends on all the vibrational modes as well as the coupling between them. In Table VIII, certain values pertaining to the MMPT surface are compared with the CCSD(T) PES.^12^ Although the MMPT-barrier height is slightly too high compared with the CCSD(T) reference calculations, the effective barrier is lower by 225 cm^–1^, which will affect the computed tunneling splittings in a fully dimensional nuclear dynamics treatment, as tunneling through low effective barriers is enhanced. The same effective barrier appears in the Eyring expression for the rate constant and thus, rates will also be predicted too high when using this PES. This error is mostly canceled out when taking the ratio of results to obtain the isotope effect and thus, KIEs determined on the MMPT PES may be expected to be quite reasonable. This is because the shape of the H transfer barrier itself is well described with MMPT.

**TABLE VIII.** Malonaldehyde PES data (in cm^–1^) comparing MMPT with the CCSD(T) PES.^12^ The effective barrier is given by the actual barrier height plus the difference in the harmonic zero-point energies (ZPE) between the transition state (TS) and minimum (min).

Method	Barrier	ZPE (min)	Imag. freq.	ZPE (TS)	Effective barrier
MMPT	1517	14 255	1463	12 970	232
*ab initio*	1430	14 853	1300	13 980	557

The tunneling splitting was previously investigated using a reduced dimensionality harmonic bath averaged Hamiltonian (HBA) together with interactions computed at the CCSD(T) level of theory.^116^ Such a treatment yielded an effective reduced mass of the transferring hydrogen atom by reproducing the experimentally determined tunneling splitting. Using isotope relationships for the transferring D-atom yielded a D-splitting of 2.9 cm^–1^, which favorably compares with that from experiments (2.915 cm^–1^).^104,105,117^ Using the same effective masses together with the MMPT PES gives splittings of 36.1 cm^–1^ for hydrogen and 5.8 cm^–1^ for deuterium (i.e., an isotope effect of 6.2).^116^

Contrary to the results of this low-dimensional treatment, fully dimensional ring-polymer instanton calculations using the same MMPT-PES yield tunneling splittings of 121 cm^–1^ and 13.9 cm^–1^, i.e., an isotope effect of 8.7. Although the splittings are about a factor of 5 too large (and a factor of 3 larger compared with the HBA treatment), the isotope effect is fairly close to the experimentally observed value of 7.4. The origin for overestimating the tunneling splitting but still reasonably well describing the isotope effect is due to the effective barrier being too low as discussed above. It is known from previous studies that H-tunneling splittings are exquisitely sensitive to the shape of the PES and to the number of degrees of freedom explicitly treated in the dynamics.^52^ For example, in a fully 21-dimensional instanton study using on-the-fly computation for the PES, the values for H-transfer range from 4.5 cm^–1^ (QCISD) to 77 cm^–1^ (MP2), whereas for a planar (15 dimensional) treatment, they range from 8.1 cm^–1^ to 170 cm^–1^.^118^ Even though the out-of-plane vibrational modes are not displaced along the instanton pathway, the change in their frequencies plays a large role in determining the tunneling splitting. This is related to the effective ZPE-corrected barrier height, which determines the classical TST rate.

Therefore, one concludes that in order to build a model of a proton transfer which will lead to accurate predictions for the tunneling splitting, it is not enough to ensure that the barrier height is correct. All modes contribute to the tunneling splitting if they are coupled to the proton transfer and it is necessary to ensure that the frequencies of these modes are correct at both the minimum and transition state. Exactly the same conclusions are true of building models to describe rates as seen from the dependence of Eyring TST on the zero-point-energy corrected barrier height.

### A. Kinetic isotope effect from path-integral simulations using the MMPT force field

Using this MMPT potential, a quantum mechanical treatment of the kinetic isotope effect of the thermal rate constants in MA was attempted. The KIE for the intramolecular hydrogen transfer in MA has not been determined experimentally and is probably not directly observable, as it is a symmetric unimolecular reaction in the gas-phase. Combining a fully dimensional and validated PES^116^ based on MMPT^110^ with a quantum instanton^8^ path-integral Monte Carlo (PIMC) simulation, the primary H/D KIE in MA was found to be 5.2 ± 0.4 at room temperature.^119^

Table IX presents the KIEs from PIMC and classical transition-state theory. The KIE from Eyring TST describes the effects from the quantization of vibrations within a harmonic approximation, and the KIE from Wigner also includes the effect of tunneling on the top of this via Eq. (2). It is seen that tunneling effects are not as important as that of quantizing the vibrations. This is confirmed by separately considering the contribution of ZPE to the KIE within a more accurate anharmonic simulation, which typically captures 90% to 95% of the total KIE.^119^ Furthermore, the contributions of translations and rotations to the KIE effect are negligible over this temperature range. PIMC also includes tunneling effects and anharmonic vibrational quantization and thus is expected to be the most accurate of the methods. It finds a smaller KIE compared with Eyring and Wigner TST probably because of anharmonic effects, and suggests that Wigner's approximation is over predicting the tunneling effect here.

**TABLE IX.** KIEs for MMPT malonaldehyde.

*T*/K	Eyring	Wigner	QI
1500	1.62	1.69	1.07 ± 0.08
750	2.25	2.56	1.42 ± 0.07
500	3.25	4.09	2.3 ± 0.1
375	4.71	6.52	3.6 ± 0.2
300	6.86	10.21	5.2 ± 0.4
250	9.98	15.70	5.5 ± 0.5

### B. Simulations of the kinetic isotope effect on a zero-point-energy-corrected potential-energy surface

**FIG. 3.**
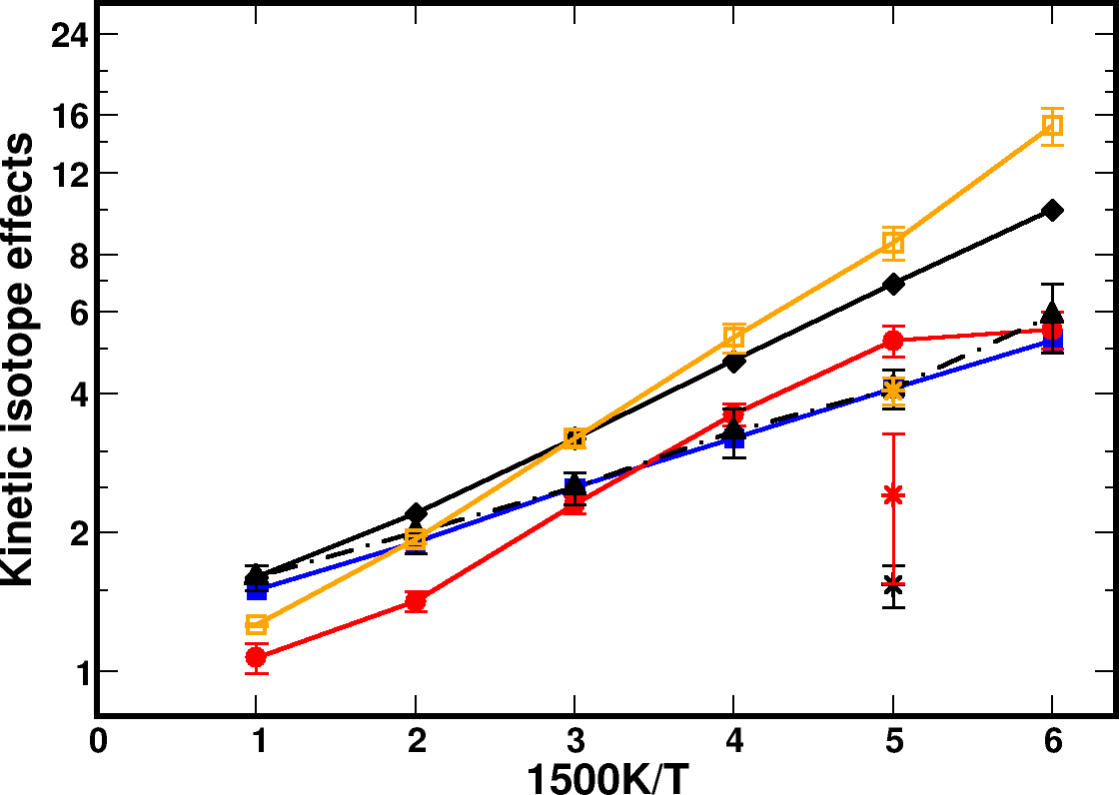
Temperature dependence of the KIE of malonaldehyde by using different methods: (1) conventional transition-state theory using the MMPT force field (black solid line with diamonds) and MP2 calculations (blue with filled squares);^119^ (2) quantum instanton path-integral Monte Carlo simulations (red solid line with circles); (3) MD simulations using the MMPT force field with ZPE corrected PT potential (black dot-dashed, this work) and (4) PI-BQCP (orange with open squares, this work). KIEs from Ref. 120 at *T* = 300 K are given as single asterisks which correspond to results obtained by using transition-state theory (TST, black), path-integral quantum transition-state theory (PI-QTST, orange) and quantum instanton (QI, red) methods using a different potential energy surface.

The PIMC simulations have confirmed that ZPE-effects play the dominant role for a realistic description of the KIE in MA, whereas tunneling contributions are insignificant over the temperature range considered. Building on this, the MMPT PES was corrected for zero point effects, and classical molecular dynamics (MD) simulations were run at different temperatures for H- and D-transfer. For including zero-point effects into classical MD simulations with the MMPT force field, a point-wise vibrational correction to $V_{0}(R,\rho )$ was carried out. For this, the normal modes at each point (R,ρ) (R∈[2.2,3.2] Å and ρ∈[0.8,R−0.8] Å, see the definition of *ρ* in Sec. IV A, in increments of 0.1 Å and 0.05 Å, respectively) were calculated. Then, the sum of zero-point energies was added to $V_{0}(R,\rho )$ at the grid points. This yields a new PES, which can be used in classical MD simulations, which were carried out in the gas phase at 250 K to 1500 K with CHARMM.^121–123^ Heating and equilibration simulations were run for 60 ps in total with a time-step of Δt=0.1 fs. Then, 50 ns of *NVE* production runs were followed and geometries were collected every 1 fs for analysis. For calculating the hydrogen transfer (HT) and deuterium transfer (DT) reaction rates, cumulative hazard plots were employed.^124,125^ All results were averaged over 10 independent MD simulations. The results of these calculations are reported in Fig. 3, see black, dot-dashed line.

The computed rate constants for HT and DT from MMPT-MD simulations on the ZPE-corrected PES are given in Table X. For the ZPE-corrected PESs, the barriers for H- and D-transfer are 2.1 and 2.8 kcal/mol, respectively, compared with a barrier of 4.3 kcal/mol on the original, uncorrected MMPT PES. The simulations yield HT reaction rates ranging from 20 ns^–1^ to 2.6 × 10^3^ ns^–1^ for temperatures between 250 and 1500 K. As expected, the DT reaction rates are lower than those for HT reactions and range from 3.3 ns^–1^ to 1.6 × 10^3^ ns^–1^ at the respective temperatures. The previous study calculated the rates for HT/DT in malonaldehyde by using the conventional transition-state theory (CTST).^119^ For a comparison, CTST gives the rates of 540 ns^–1^ and 80 ns^–1^ for HT and DT reactions at 300 K, respectively, which are around 11- and 7-fold larger than those from the rates obtained by MD simulations on the ZPE-corrected PES. Clearly, the results from CTST on the bare PES overestimate accurate KIEs from path-integral Monte Carlo simulations.

**TABLE X.** The KIE for MA from MD simulations on the ZPE-corrected MMPT PES and the path-integral simulation with bisection quantized classical path approach (PI-BQCP^126^) using the uncorrected MMPT potential. For MD-MMPT, rate constants are given for both HT and DT transfer (in 100/ns units), whereas for PI-BQCP method, the free energies of activation (in kcal/mol) are reported. The statistical errors are given in parentheses and refer to the last digits for each calculated value.

*T*(K)	MD-MMPT			PI-BQCP		
	$k_{H}$	$k_{D}$	KIE	$\Delta F_{H}^{‡}$	$\Delta F_{D}^{‡}$	KIE
1500	26(1)	16(1)	1.6(1)	4.581(11)	5.281(6)	1.26(1)
750	9.6(3)	4.7(2)	2.0(2)	2.851(12)	3.828(5)	1.93(4)
500	3.0(1)	1.2(1)	2.5(2)	1.987(17)	3.144(9)	3.20(15)
375	1.2(1)	0.35(3)	3.3(4)	1.439(17)	2.680(9)	5.29(38)
300	0.47(1)	0.12(1)	4.1(4)	1.018(15)	2.293(10)	8.48(69)
250	0.20(1)	0.033(4)	5.9(10)	0.624(13)	1.976(8)	15.2(14)

Correcting the PES for zero-point energy effects and using classical MD simulations for determining the HT and DT rates yields quite favorable agreement of the KIE with quantum simulations, see red circles and black triangles in Fig. 3. Thus, for KIEs, such a zero-point corrected PES can be a computationally attractive and quantitatively meaningful alternative to high-dimensional, CPU-intensive PIMC simulations. This is contrary to recent results for elastic and inelastic collisional rates for $N_{2}^{+}$–Ar for which a ZPE-corrected PES gives rates for rotational excitation considerably larger than those measured experimentally, even with a high-level, fully dimensional CCSD(T) PES.^127^

Finally, the path integral with bisection quantized classical path sampling approach (PI-BQCP) was used together with the MMPT PES (without ZPE correction).^126^ PI-BQCP consists of a (classical) umbrella sampling simulation and a post-processing step to include quantum effects. For each temperature (250 K to 1500 K), heating and equilibration simulations were run for 20 ps, followed by an additional 50 ps equilibration with a harmonic constraint [50 kcal/(mol⋅Å^2^)] applied to the progression coordinate of *r*_1_–*r*_2_ (see Fig. 2, 37 windows covering the interval from –0.9 to 0.9 Å). *NVT* production simulations were run for 1 ns and structures were recorded every 10 ps. For the path-integral simulations, only 3 atoms in the PT motif ($O-H^{*}\cdots O$) were quantized with 256 beads for both HT and DT reactions. KIEs were then obtained by calculating the quantum corrections to the free energies of activation

$$k_{\text{HT}}/k_{\text{DT}}=e^{-\beta (\Delta F_{H}^{‡}-\Delta F_{D}^{‡})}.$$ (6)

Further technical details for the PI-BQCP method can be found in Ref. 126. The results from PI-BQCP simulations are also reported in Fig. 3 (solid, orange trace) and in Table X. Compared with PIMC simulations, the PI-BQCP simulations overestimate the KIE by about a factor of three at low temperatures, but capture the high-temperature results quite well. In fact, they are closer to the PIMC results than classical MD simulations on the ZPE-corrected PES at 1500 K. However, for lower temperatures, PI-BQCP is less suitable as judged by the PIMC reference calculations and using classical MD on a ZPE-corrected PES appears to be more meaningful.

Overall, the results presented here suggest that classical MD simulations on a zero-point-energy-corrected PES yield quite favorable agreement compared with PIMC simulations on the uncorrected PES. Contrary to that, PI-BQCP simulations rather follow the Wigner-results (see Table IX) and overestimate the KIE, particularly at low temperature. Hence, when tunneling is not expected to be important, classical MD simulations on a zero-point-energy-corrected PES may be a computationally advantageous alternative to full quantum dynamics simulations for estimating the temperature dependence of the KIE.

## V. THE AZZOUZ-BORGIS MODEL FOR PROTON TRANSFER IN POLAR SOLVENT

The Azzouz-Borgis model^14–16^ for proton transfer reaction in liquid methyl chloride is an important system, which has been extensively used to test behavior of different methods for calculating reaction rates in solution.^128^ Both the quantum instanton approximation^129^ and ring-polymer molecular dynamics^130^ have been applied to this problem. Their results are presented in Table XI along with reaction rate constants obtained by classical TST and the full classical rate including dynamical recrossing. The semiclassical instanton approach is not applicable to reactions in solution, as there would be a very large number of slightly different instanton trajectories for each configuration of the solvent. The only practical way to overcome this would be to sample over the different instantons which is effectively what is done by QI.

**TABLE XI.** Rate constants for transfer of hydrogen and deuterium in the Azzouz-Borgis model, as well as the corresponding kinetic isotope effect. Rates are given in units of 10^10^ s^–1^.

method	$k_{H}$	$k_{D}$	KIE
TST^a^	1.43 × 10^–3^	⋯	⋯
Classical^a^	1.90 × 10^–4^	⋯	⋯
QI^b^	17	0.36	47
RPMD^c^	1.7	0.075	23

^a^ Reference 131.

^b^ Reference 129.

^c^ Reference 130.

Both QI and RPMD predict a much faster rate than that obtained from classical theories. Thus, it is clear that quantum effects and especially tunneling are very important for this system. The quantum instanton approximation, however, predicts rates much larger than RPMD. The authors of Ref. 130 speculate that the difference could be caused by recrossing effects, as ring-polymer molecular dynamics approach allows to at least partially account for them, while other quantum transition-state theories do not. A similar effect is seen in the classical rate which, as for RPMD, is reduced by about a factor of 10 due to recrossing.

No exact benchmark rate exists for this system, although multi-layer MCTDH calculations have been performed on a system-bath model designed to emulate this reaction.^131,132^ The rate constant obtained from this procedure was 9.3 × 10^10^ s^–1^, which is in better agreement with QI than RPMD. The reasons for this remain unclear, although one possible cause of the discrepancy with RPMD could be that the mapping to a linearly coupled harmonic model cannot describe the intricacies of the full system.

## VI. DISCUSSION

We have reviewed several methods for describing kinetic isotope effects for proton transfers in bimolecular and unimolecular systems. Depending on the efficiency and accuracy required, each method has a domain of validity. In particular, the Wigner-Eyring approach can give fairly accurate predictions for high temperature KIEs based only on a single-point and frequency calculation. However, this method does not describe tunneling correctly and in order to obtain accurate KIEs at low temperature, it is necessary to use a fully dimensional quantum method, the simplest of which is semiclassical instanton theory. This gives accurate rates, tunneling splittings, and KIEs below the crossover temperature. However, in its standard form, it cannot be applied at higher temperatures and exhibits an error near crossover. Work is in progress to extend and improve the SCI approximation to overcome this problem.^31^

The QI approach is particularly well designed for studying KIEs. Recent improvements in the efficiency of the path-integral description speed up convergence significantly. This method remains more computationally expensive than SCI, but has been found to be more accurate in most cases where the statistics can be converged. This is because it does not have the problem at crossover and also treats the anharmonicity of all modes explicitly.

Due to the efficiency of the SCI method, it can be applied using an on-the-fly calculation of the potential-energy surface. We have compared these results with those computed using a fitted PES and found that they deviate from each other at low temperatures. This is because as the temperature is lowered, the instanton explores a wider region around the barrier top and is thus more susceptible to errors in the fit. In fact, we expect that the error due to a fitted surface could have a larger effect on the rate than the approximation inherent in the instanton method.

The tunneling splitting calculations which we performed concur with previous studies^52,118^ that an accurate multidimensional description of the PES is necessary to obtain results in agreement with experiment. In particular, the tunneling splitting is not purely described by a one-dimensional barrier along an effective reaction coordinate, but all the vibrational modes of the system have the potential to contribute. For this reason, the comparison of the predicted tunneling splitting with the experimental observation gives a very strong test of the accuracy of the PES,^133^ not just of the barrier height but also the barrier shape and the coupling between vibrational modes. The same analysis holds for calculations of rate constants.

Therefore, when building a force field to describe proton transfer, it is important to ensure that the coupling between the vibrational modes is correctly described. However, the isotope effect itself is less sensitive and can be obtained fairly accurately by the MMPT force field and semiclassical instanton theory. At higher temperatures, the QI approach can be used, but a cheaper alternative gives similar results by running classical dynamics on a zero-point-energy corrected PES.
